# Challenges for Lower-Middle-Income Countries in Achieving Universal Healthcare: An Indian Perspective

**DOI:** 10.7759/cureus.33751

**Published:** 2023-01-13

**Authors:** Meenu G Sharma, Harvinder Popli

**Affiliations:** 1 School of Pharmaceutical Sciences, Delhi Pharmaceutical Sciences and Research University, New Delhi, IND

**Keywords:** universal health coverage, lower middle income countries, healthcare system, public health, india

## Abstract

Universal health coverage (UHC) by 2030 is a commitment of the global community adopted as Sustainable Development Goal 3.8. UHC, as defined by WHO, means all people have access to quality health services, when and where they need them, and without financial hardship. However, low-income and lower-middle-income countries, faced with competing priorities, find themselves struggling to muster enough resources to steer towards this goal at the desired pace. India is the largest lower-middle-income country, accounting for almost 18% of the world’s population. How it performs in moving towards this goal will have a significant impact on achieving UHC at a global level. India has witnessed noteworthy improvement in several health indicators and the UHC service coverage index in recent decades, but the progress on improving service capacity and access has been rather slow given the enormity of its population, scarcity of funds, grossly inadequate public infrastructure, shortage of trained workforce, disparate needs of various regions of the country, lack of healthcare system integration, changing disease demography, and slack regulatory framework. The recent push through National Health Mission aims to address some of these challenges; however, a fragmented health delivery structure ossified over decades has been slow to keep up with the requirements of the country's massive and diverse population. This paper discusses the inherent characteristics and key challenges faced by the healthcare delivery system of India in achieving UHC.

## Introduction and background

India is a country of diversities, not just in languages, terrain, food, attire, culture, and belief systems, but also in healthcare needs and the infrastructure to address these needs. These diversities that bring vibrancy to society also carry with them the administrative challenges in devising and implementing healthcare solutions. India is committed to achieving universal health coverage (UHC) by 2030 as enshrined in Sustainable Development Goals (SDG) adopted by 193 countries in 2015 [1]. The path to UHC has been challenging, with dichotomies of an ambitious upwardly mobile lower-middle-income economy, offset by the inherent complexities of the enormous size of the population to cater to, fragmented governance mechanism for health, and the fundamental healthcare delivery structure hardened over decades.

India has been witnessing fast-paced economic growth, fueling aspiration and opportunity for the upliftment of living standards and the well-being of its citizens. A significant proportion of its population has been lifted out of poverty in recent decades, reducing the population living in multidimensional poverty from 55.1% in 2005-2006 to 27.9% in 2015-16 and further to 6% in 2021 and lifting 450 million out of poverty in the process [2,3].

Given the pace of economic growth and shrinking poverty levels, it is natural to expect similar trends in the healthcare scenario as well. Some health indicators have indeed shown significant progress. For instance, life expectancy improved from 62·5 years in 2000 to 69.9 years in 2020 [4]; the infant mortality rate declined significantly from 67 per 1000 in 2000 to 27 per 1000 live births in 2020 [5]; the maternal mortality ratio decreased from 370 per 100,000 livebirths in 2000 to 145 per 100,000 livebirths in 2017 [6]; HIV/AIDS spread has been controlled [7]; over two billion COVID-19 vaccine doses have been administered [8]; and the country has achieved polio-free [9] and maternal/neonatal tetanus-free status [10]. The age-standardized DALY rate in India dropped by 36% from 1990 to 2016, indicating overall progress in reducing disease burden, but this gain has been accompanied by an epidemiological transition to non-communicable diseases that now contribute to about 55% of the disease burden and over 60% of deaths [11]. Overall, ischemic heart disease, chronic obstructive pulmonary disease, depression, hemorrhagic stroke, and diabetes are among the leading non-communicable causes of the country’s disease burden, while infectious diseases still remain a drag on the system. This changing disease demography is an additional challenge that the country needs to consider on its path to achieving UHC.

Recently increased focus on the overall hygiene and sanitation infrastructure is also a step in the direction of improving the health of the population. With only around 40% population having access to clean toilets in 2014, India has invested heavily in the “Clean India” campaign and the country was declared open-defecation-free in October 2019 [12]. A similar campaign was started in 2019 for providing piped drinking water to every household, taking the proportion of the population having tap water from 16.7% in 2019 to approximately 55% by November 2022, with a target of 100% by 2024 [13]. These hygiene initiatives are expected to have a long-term impact in reducing the disease burden of infectious diseases, especially diarrhea, which is still one of the leading causes of mortality in India.

It is well acknowledged that sustaining the growth of a country is possible only when healthcare is accessible to all its citizens without causing undue financial burden. Successive governments in the center and at the state level have been making concerted efforts to bring health to the forefront of the political discourse. Schemes such as Ayushman Bharat (Sanskrit for “long live India”) have been rolled out to provide financial protection (PM-JAY) to the bottom 40% of the population for accessing tertiary care and simultaneously strengthening the primary care infrastructure by setting up 150,000 health and wellness centers (HWCs) across the country [14]. However, faced with the challenge of serving a huge population with diverse needs vs. the grossly inadequate resources available, the infrastructure is struggling to keep up with the ambitious goals the country has set for itself.

## Review

Indian healthcare system: construct and layers

Healthcare delivery in India is provided both by the public and the private sectors with a significant skew towards private players, driven by inadequacies of infrastructure in the public setup. Private facilities provide healthcare access for about 70% of outpatient visits and 60% of hospital admissions [15]. India has approximately 1.9 million hospital beds, with a disproportionate concentration in the private sector [16]. Diagnostics setups and retail pharmacies are almost entirely in the private sector as well. This skew creates pressure on financing the healthcare sector, which is evidenced by the fact that only 27% of the current healthcare expenditure is borne by the government whereas 73% comes from private financing including a fairly large proportion of out-of-pocket-expense (OOPE) of over 53% [17].

**Figure 1:**
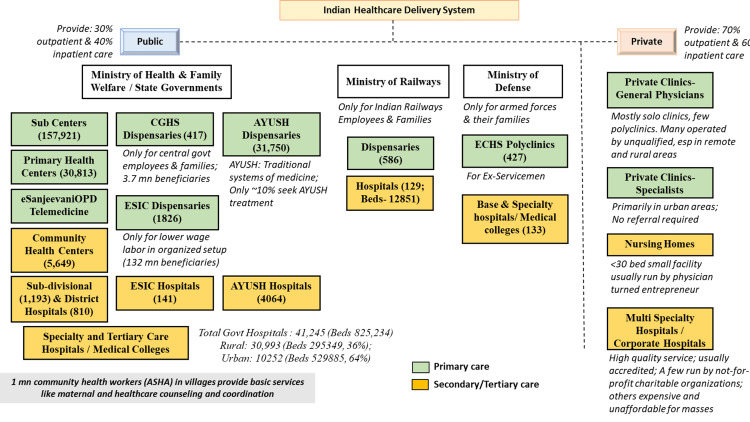
Overview of healthcare providers in India Govt hospital numbers also include autonomous hospitals set up and funded by central and state governments References: [15], [17-18], [20-23] ASHA: Accredited Social Health Activist; AYUSH: traditional and non-conventional systems of healthcare and healing which include Ayurveda, Yoga, Naturopathy, Unani, Siddha, and Homoeopathy; CGHS: Central Government Health Scheme; ECHS: Ex-servicemen Contributory Health Scheme; ESIC: Employees’ State Insurance Corporation (Contributory Insurance Scheme)

In terms of the public health system, India has a tiered structure (Figure 1) for making healthcare delivery accessible to its massive population spread across an expansive geographical area. At the base of the public health system is a vast network of 156,101 and 1,718 Sub Centers (SCs) in rural and urban India respectively, each serving a population of 3,000-5,000 and 25,140, and 5,439 Primary Health Centers (PHCs) each serving 20,000 to 30,000 individuals [18]. These SCs and PHCs are the points of first contact for patients seeking medical care. Of note, 150,000 SCs/PHCs are being upgraded/transitioned into more holistic Health and Wellness Centers (HWCs) with an expanded basket of services [19]. This system of primary healthcare is complemented by Community Health Centers (CHCs), district-level hospitals, and tertiary hospitals for complicated diseases and those requiring inpatient care. There are 5,481 and 470 CHCs catering to 100,000 persons in rural and urban India respectively coupled with 1,224 Sub Divisional/District and 764 District Hospitals across the country catering to both rural and urban areas [18]. In addition to the physical infrastructure, the central government has also introduced a national teleconsultation service, eSanjeevaniOPD. Launched in April 2020, this service has provided over 87 million tele-consults in less than three years and may bridge the access gap to some extent for primary care services in the future [20]. Much of the public health infrastructure is managed under the National Health Mission with an aim to strengthen healthcare delivery in both rural and urban areas through various national health programs for improving reproductive-maternal-neonatal-child and adolescent health and management of communicable and non-communicable diseases [21]. The private healthcare sector is heterogeneous both in demographics with respect to states, rural and urban locations, and also in the quality and distribution of health providers, especially specialists and surgeons, the majority of whom practice in urban centers only, requiring the rural populations to commute long distances to access such healthcare facilities. There is no reliable data on the number of private clinics in India that are the key providers of primary care. Often such clinics in remote and rural areas are run by unqualified persons.

Indian healthcare system: financing

India spends about 3% of its GDP on health while the global average is about 10% (2019) [17,24]. Of this, the government expenditure is roughly 1.3% of GDP (vs. 6% global average) or about 40% of the total healthcare expenditure. Of note, 54.4% of total health expenditure is borne by households of which 48.2% is OOPE, which is equivalent to 53.2% of current healthcare expenditure (total expense minus capital expenditure) and 6.2% is the cost of private insurance by the households [17].

The penetration of insurance remains low at 37% of the population (Table 1) [25] despite the recent push through the PM-JAY scheme. PM-JAY plans to cover the poorest 40% of the population by covering the cost of treatment at empaneled tertiary care private hospitals, with coverage in November 2022 reaching 14% of the population [26]. Private insurance penetration is below 4% due to the high cost of insurance and low acknowledgment of risk. Over 66% of all lives covered or 25% of the population is covered by government-sponsored schemes [25] that primarily extend to the lowest income strata of the population, leaving the middle class with the highest exposure to OOPE.

**Table 1:** Penetration of health insurance in India, 2020-2021 PM-JAY: Pradhan Mantri Jan Arogya Yojana

	Lives covered (million)	% Population	Coverage
Government-sponsored schemes	342.9	24.8%	Variable; PM-JAY, the largest government-sponsored scheme covers hospitalization expenses up to INR 500,000 per family per year; state-sponsored schemes may have a lower cover. Govt. employees and ex-employees, armed forces personnel, and ex-servicemen are covered, along with their families, by the government with negotiated rates at private health facilities and unlimited coverage for the beneficiary. May be merged with PM-JAY in the future
Employer-sponsored group hospitalization insurance	118.7	8.6%	Variable; usually in the range of INR 300,000 to 500,000 per family per year; premium negotiated by employers, cost usually counted in perks for the employee
Privately funded individual insurance	53.1	3.8%	Variable; based on the premium-paying capacity of individual since premiums are very high; exclusion of pre-existing diseases and senior citizens is common
Total lives covered	514.7	37.3%	All health insurance policies only cover the cost of hospitalization. During 2020-21, insurers settled 14 million health insurance claims valued at INR 430 billion, with an average of INR 31,000 (USD 385) per hospitalization

All citizens can access free outpatient and inpatient care at public facilities with no co-payments or deductibles. Essential medicines and diagnostic tests are generally free in the public setup, but other medicines need to be purchased from private pharmacies at full cost, as also those essential medicines which are not available. Despite a mandated list of essential medicines that should be available at PHCs and sub-centers across the country, frequent non-availability of some or several such medicines has been reported, possibly due to low budget allocation for medicines, inability to forecast needs accurately, and inefficient drug supply chain management [27]. The central government is setting up low-priced generic medicine pharmacies known as Jan Aushadhi outlets that sell drugs at hugely discounted prices in the private sector, sometimes with 70-80% discount over branded generic versions of the same drug [28]. Private health facilities are expensive and fully paid for by most patients, except those covered under some government scheme or private health insurance (Table 2). Since more than 70% of outpatient care (72% in the rural areas and 79% in the urban areas) and more than 60% of inpatient care (58% in rural areas and 68% in urban areas) are provided by the private sector [29], the overall financial burden on the common population is very high.

**Table 2:** Cost and financing of treatment at public and private facilities in India OOP: out-of-pocket

	Public facilities	Private facilities
Primary care consult	Free	Variable (not regulated, all OOP)
Specialist consult	Free	Variable (not regulated, all OOP, can be very high)
Hospitalization	Free; some associated costs, e.g., drugs may need to be paid OOP	Variable (not regulated except the negotiated rates for government-sponsored schemes); 63% of the population does not have any insurance coverage and all expenses paid OOP
Prescription drugs (outpatient)	Essential medicines; free but availability not certain; others procured OOP	All OOP, several drugs regulated by a ceiling price
Diagnostics	Free	Variable (not regulated, all OOP)
Medical devices	Variable (not regulated, mostly OOP)	Variable (not regulated, mostly OOP)

Performance of lower-middle-income countries and India on UHC indicators

The World Bank classifies countries into four income groups: low, lower-middle, upper-middle, and high income based on the gross national income (GNI) per capita of the previous year. Lower-middle-income countries are those with GNI per capita between $1,085 and $4,255, representing a diverse set with wide variation in size, population, GDP, sociopolitical landscape, and health needs. However, being resource-constrained, yet ambitiously upward mobile at the same time, the majority of these 53 countries still struggle to provide basic services such as electricity, water, education as well as quality health services to its citizens. In terms of healthcare, this bloc of countries has made tremendous progress in their path to achieve UHC, showing the largest increment in the service coverage index (SCI) over the last decade [4,5], which is the official indicator for monitoring access to healthcare. A 13-point increment for India and an 11-point average increment for all lower-middle-income countries in SCI scores have been witnessed in the last decade. The most striking improvement has been in the control of infectious disease, significantly improving the sub-index, driven by a concerted focus in the recent past, after the earlier push for reproductive, maternal, newborn, and child health (RMNCH) provision. However, the service capacity and access sub-index is still visibly sub-par with the score still much lower than the desirable >80 level, pointing to the extreme hurdles being faced by India and other similar economies on this front (Tables 3, 4).

**Table 3:** Key UHC indicators for World Bank income groups and India NCD: non-communicable diseases; RMNCH: reproductive, maternal, newborn, and child health; SCI: service coverage index; UHC: universal health coverage

	UHC service coverage index (SCI)	UHC service coverage sub-index score				SCI score increment vs. 2010	
		RMNCH	Infectious diseases	NCD	Service capacity and access	Overall SCI	Infectious diseases sub-index
Global	67	76	70	62	67	9	26
Low-income	42	52	43	69	23	5	17
Lower-middle-income	58	68	62	62	44	11	32
Upper-middle-income	77	84	77	60	89	9	29
High-income	83	89	88	64	95	3	9
India	61	72	71	63	44	13	41

**Table 4:** Key financial and capacity indicators for World Bank income groups and India CHE: current healthcare expenditure; GDP: gross domestic product; GNI: gross national income; OOPE: out-of-pocket expenditure

	GNI per capita (USD)	CHE per capita (USD)	CHE as % of GDP	Govt expenditure on CHE as % of GDP	OOPE as % of CHE	Hospital beds per 10,000	Physicians per 10,000
Global	12,070	1,122.0	9.8	5.9	18.0	28.9	17.6
Low-income	719	34.9	4.9	1.1	43.2		3.2
Lower-middle-income	2,428	96.6	3.8	1.4	48.2	7.7	8.5
Upper-middle-income	9,636	276.6	5.8	3.2	32.2	38.9	23.4
High-income	45,605	5,638.7	12.5	7.7	13.6	52.8	37.3
India	2,170	63.7	3.0	1.0	54.8	5.3	9.3

Most other lower-middle-income countries also demonstrate similar profiles on UHC indicators and even the increments in recent years show similar trends. Table 5 summarizes the progress of other lower-middle-income countries in Southeast Asia on the SCI index and sub-indicators [4,5]. Indonesia and Bangladesh are the next two countries in this group in terms of GDP and population size and both show a similar pattern of progress in controlling infectious diseases, increasing the burden of non-communicable diseases (NCDs) with relatively lower improvement in management, and lagging in the service capacity, although Indonesia has made significant progress on this front recently after the introduction of a comprehensive UHC policy in 2014.

**Table 5:** Key UHC indicators for other lower-middle-income countries in Southeast Asia GDP: gross domestic product; GNI: gross national income; NCD: non-communicable diseases; RMNCH: reproductive, maternal, newborn, and child health; SCI: service coverage index; UHC: universal health coverage

	GNI per capita (USD)	Population (million)	GDP (USD billion)	UHC service coverage index (SCI)	UHC service coverage sub-index				SCI score increment 2019 vs. 2010
					RMNCH	Infectious diseases	NCD	Service capacity and access	
Indonesia	4,140	276	1,186	59	59	51	53	53	14
Bangladesh	2,620	166	416	51	51	46	56	44	14
Philippines	3,640	111	394	55	55	62	66	32	12
Vietnam	3,560	98	363	70	70	70	69	61	11
Pakistan	1,500	225	346	45	45	36	51	35	9
Sri Lanka	3,820	22	85	67	67	66	62	63	12
Myanmar	1,140	55	65	61	61	77	51	49	16
Nepal	1,230	30	36	53	53	60	58	30	16
Cambodia	1,550	17	27	61	61	70	73	37	8
Lao PDR	2,520	7	19	50	50	62	65	26	11
Bhutan	2,840	1	2	62	62	63	47	61	13

Challenges faced by the Indian healthcare system

Population and Changing Disease Demography

**Figure 2:**
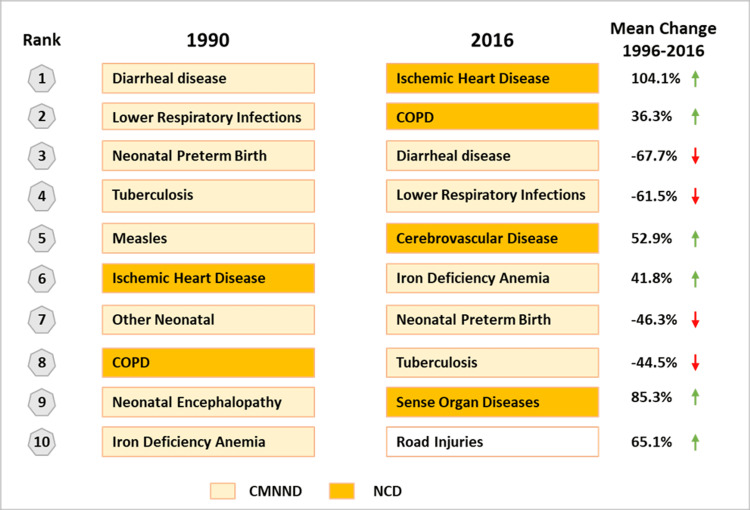
Top 10 causes of disease burden (DALYs) in India, 1990 vs. 2016 CMNND: communicable, maternal, neonatal, and nutritional diseases; COPD: chronic obstructive pulmonary disease; DALY: disability-adjusted life year; NCD: non-communicable diseases

With a population of 1.39 billion, India is the second most populous country in the world and home to about 17.7% of the global population. Unlike China's, India's population is expected to continue to grow, surpassing 1.5 billion by 2030 when the country would become the most populous in the world. A massive population with more than 2,000 ethnic groups and diverse living conditions [11] makes the challenges of scarce healthcare resources even more acute. Driven by improving life expectancy and changing lifestyles, the disease profile of the country is also showing a rapid transition. The top five individual causes of disease burden in India in 1990 were communicable, maternal, neonatal, and nutritional diseases (CMNNDs), whereas, in 2016, three of the top five causes were NCDs (Figure 2). NCDs now contribute about 62% to the overall mortality in India with cardiovascular diseases being the largest cause of NCD-related deaths. In 2016, 27.5% of deaths were due to CMNNDs, 61·8% due to NCDs, and 10·7% due to injuries [11].

A system that has been designed for decades to deal with CMNNDs and is not quite finished fighting the battle is now required to also add components for NCDs that have distinct requirements in terms of approach, infrastructure, and strategies. NCDs need to focus on screening, preventative care, and long-term management, requirements that India’s primary health infrastructure is ill-equipped to handle. There is a recognition of the need to incorporate this capability in the redesigning of PHCs into HCWs; however, with little bandwidth to deviate from the current course of tackling communicable diseases, the focus on NCDs is likely to remain diluted, creating long-term challenges of severely increased health burden on the already inadequate healthcare system.

Social Inequality

Access to healthcare in India is strongly influenced by socioeconomic factors of not only gender and income level but also caste, social status, and geography. For example, life expectancy in urban areas is higher than in rural areas with a gap of 4.8 years [30]. Similarly, the more affluent (top 20%) live 7.6 years longer than the poor (bottom 20%) [30]. Of note, 73% of the public hospital beds are in urban areas in India even though 69% of people live in rural areas, putting the rural population at a great disadvantage in terms of access [29].

Health-seeking behavior is controlled by the income level and social identity of the users and service providers [31]. Since private facilities are unaffordable for a vast majority of the population, more so in rural areas, public facilities become the first point of contact as well as centers for advanced treatment for the less affluent. Public facilities are overburdened and unable to offer quality services due to deficiencies and inefficiencies of infrastructure. Hence, the lower socioeconomic groups and other vulnerable segments are left to face the challenges of access and affordability much more than the middle-class or higher-income groups [30].

Infrastructure Deficit and Disparity

Grossly inadequate infrastructure and a shortage of trained health workforce continue to remain among the greatest challenges in India’s efforts to provide healthcare to all, complicated further by inequitable distribution of these resources. In the federal structure of India, health is a governance subject managed by states, leading to heterogeneity in infrastructure, policies, access strategies, and population coverage among different states. Despite the presence of a wide network of primary healthcare centers, it is estimated that there is a shortage of 29% in PHCs and 35% in CHCs in rural India. This shortage is further accentuated in some states such as Bihar where the shortfall is 47% and 66% respectively and Jharkhand with a shortfall of 74% and 38% respectively. On the other hand, states like Kerala and Himachal Pradesh do not have any such shortfall [18].

Further, all levels of the system are plagued by a severe shortage of general physicians and specialists. Only 11% of SHCs, 16% of PHCs, and 16% CHCs meet the Indian Public Health Standards [14]. Of note, 9.6% of PHCs do not have a doctor, 33.4% do not have a lab technician, and 23.9% operate without a pharmacist. At the CHC level, 68% of sanctioned posts for specialists are vacant and there is a shortage of 83.2% of surgeons, 74.2% of obstetricians & gynecologists, 82.2% of physicians, and 80.6% of pediatricians compared to the requirements of the population served [14,18]. Availability of hospital beds is another indicator where India fares poorly compared to the global average. In India, 5.3 hospital beds are available per 10,000 population, which is much below the global average of 29 beds per 10,000 population [4,32,33]. Further, even the available beds are highly concentrated in urban areas (64% of total beds), even though the majority of the Indian population resides in villages [22].

Trained Healthcare Workforce

India has 9.3 physicians per 10,000 population, much below the global average of 17.6 and the WHO-recommended minimum threshold of 10 per 10,000 population [34]. WHO recommends a threshold of 44.5 doctors, nurses, and midwives per 10,000 population as the desired density for countries to meet the health SDGs. For India, the number currently is 36.84, representing a significant gap vs. the minimum threshold [35]. On the positive side, India has 612 medical colleges (321 government and 291 private) with 91,927 undergraduate (MBBS) seats, representing a significant increase of 83% in the number of medical colleges and 121% in MBBS seats in the last decade [34]. However, this steep ramp-up in capacity is still not sufficient to address the needs of a rapidly growing population. In an effort to fill some of this gap, over 1 million ASHA (Accredited Social Health Activists; the acronym Asha means “Hope”) workers have been recruited at the village level [23], but their role is limited primarily to delivering basic maternal and child health services.

Apart from the skew in infrastructure, the available workforce is also distributed unevenly with the variation as wide as 1.1 health workers per 10,000 population in the state of Nagaland at the lowest end and 115 per 10,000 population in the state of Kerala at the highest end [35]. Of note, 77.4% of all qualified healthcare workers are in urban areas [36] where only 35% of the total population resides, leaving the rural population with minimal access to qualified healthcare professionals. Beyond the geographical concentration, there is also a sectoral bias with over 60% of doctors and 50% of nurses/midwives employed in the private sector, even though public facilities in different states report a high level of vacancies against the sanctioned posts [34]. Despite having government-mandated bonds requiring new graduates to serve between one to five years in public hospitals, mostly in rural areas, this has not solved the problem as many doctors pay the penalty to migrate to urban settings for better-paying jobs and amenities. The migration of doctors and nurses to other countries that offer a more promising financial future is also a challenge, leaving a void back home.

Another grave concern is the existence of an unqualified workforce, which is as high as 45-56% of the estimated numbers, thus reducing the actual qualified physician density to about four and total healthcare workers to between 9-16 per 10,000 population [32,36,37]. The private sector has an equal number of qualified doctors and unqualified practitioners. A greater ratio of unqualified to qualified personnel exists in less developed states, exposing the less privileged sections of society to quackery and suboptimal medical care [31].

Fragmented Healthcare Delivery

Expansion of healthcare services and extending financial protection to the poor under the National Health Protection Mission (PM-JAY) is a huge step towards UHC but is still proving to be insufficient not just due to the size and spread of the population, but also the lack of integration of the healthcare setup. Access to healthcare is highly unregulated in India. Medical care is obtained both at private and public facilities, with no linkage or continuity from one facility to the other. No standard referral protocols and paths have been established, leaving the patients to maneuver through the system on their own. India is unique in the manner any patient can freely access any facility or specialist, public or private, at will, provided they can afford it. Patients can hop from one facility to the other taking multiple medical opinions as long as they can afford the time and cost involved. On the positive side, this practice has meant that the population that can afford it does not have to wait for weeks or months for an appointment, a diagnosis, or a surgical procedure, unlike in many other countries. One can theoretically directly consult a super-specialist or get an elective procedure done within a day in a private facility or within a short time in a public facility. On the flip side, this also leads to overuse of medical care for paying patients and bursting through the seams for public facilities. This practice is also seen as a major challenge to be resolved for the new financial protection scheme under PM-JAY to succeed in the long run and to avoid misuse by patient-provider collusion for fraudulent claims and non-essential medical care [14].

The health information systems of the country are also fragmented. Multiple agencies collect health monitoring and surveillance data [29]. However, the absence of coordination among the agencies and the reconciliation of such data results in missed opportunities for actionable insights for an improved, targeted approach to identifying and addressing key gap areas in a timely manner. The Government of India has recently rolled out a national digital health mission to address this problem [38]; however, it is still in the early stages and it is difficult to predict the success of the initiative in providing meaningful and actionable recommendations for policy decisions.

Inadequate Outlay and High Out-of-Pocket Expenditure

As healthcare receives only a small share of the government budget, a considerable portion of the cost, roughly 53%, must be borne as OOPE [17], which is among the highest in the world and about triple the global average of 18%. Recent National Health Accounts (2018-19) report by the government of India indicates a downward trend in OOPE, estimated at 48.2% of total healthcare expenditure currently, down from 64.3% in 2013-2014, which is a positive sign but still much above the global average [17]. Given the ever-increasing cost of healthcare and high OOPE, 17.3% of the population in India spends more than 10% of their household consumption or income on paying only for healthcare expenses, and about 4% spend well over 25% of income, with catastrophic impact on their ability to live a life of dignity and meet their basic needs [17,39].

The high level of OOPE in India makes healthcare costs the single biggest cause of debt, pushing about 40-50 million people into poverty every year [36]. Globally, 70 million people were pushed into extreme poverty (below $1.90 per person per day in 2011 purchasing power parity terms) in 2017, while 118 million (2%) were pushed below the $3.20 per person per day poverty line [17,39,40]. India contributes a significantly large burden to such impoverishment globally, which can be addressed through effective healthcare policies and infrastructure for achieving UHC, thus breaking the vicious cycle.

Less Stringent Regulatory Oversight

Private facilities are quite heterogeneous in their character and quality and largely unregulated. Many of these private clinics or small nursing homes, especially in remote areas, are run by unqualified ‘doctors’, or in some cases unscrupulous among the trained workforce. There is a perception of overcharging and exploitation among the patients as well as concerns about malpractices, over-prescription, overuse of procedures, pharma-physician unethical practices, and irrational treatment. Even the bigger corporate-run hospitals are perceived to be fleecing patients as no restraining regulation for private facility/provider cost exists, which lets them essentially operate as a free economy profit-making business. The high cost of operation at corporate hospitals, many of which offer plush 5-star hotel-like comfort, necessitates high charges; however, this is at odds with popular expectations of low cost for basic needs such as healthcare. There have also been concerns that doctors operating in these hospitals may be forced to put profits above patients, leading to suboptimal healthcare and an increased risk of catastrophic expenditure for the patients [41,42]. For instance, it is estimated that about 50% of all deliveries in private hospitals in India are by cesarean section as against 14% in public hospitals, while the WHO-recommended ideal range is 10-15% [43].

All hospitals are required to prominently display information on patients’ rights to know about their treatment process [44]. However, the lack of education and awareness among patients, constraints on physicians’ time in an overstretched system, and the perceived position of power represented by the doctor (life-saviors) make it difficult for most patients to question the treatment being given. Although services of doctors/hospitals are covered under consumer protection laws, the tortuous legal system that takes decades to arrive at a decision and the absence of a well-established mechanism for deciding the quantum of liability in cases of medical negligence deter patients from demanding high-quality healthcare services [45]. Furthermore, despite having a well-defined Pharmacovigilance Program of India (PvPI) and adverse drug reaction (ADR) monitoring centers across the country, there is widespread underreporting of ADRs [46]. Awareness about the pharmacovigilance system is lacking even among healthcare professionals [47], let alone patients, leading to negligible reporting by consumers of the healthcare services.

Another unique characteristic of the Indian healthcare system is the slack enforcement of laws related to dispensing prescription drugs. Any and almost all drugs are available for purchase without a valid prescription, leading to rampant self-medication. According to a recent systematic review, the pooled prevalence of self-medication is as high as 53.6% [48]. This practice has led to dangerous clinical consequences such as widespread antibiotic resistance, steroid overuse, increased risk of adverse reactions and prolonged illness, etc., consequently causing an increased burden on healthcare as well as driving up the overall cost of healthcare in the country.

## Conclusions

Lower-middle-income countries have been successful to quite an extent in managing acute conditions like perinatal care and infectious diseases, leading to improved health outcomes. However, they continue to lack resources to bolster universal access and financial protection covering their entire population. People still need to travel long distances for specialist or inpatient care for complicated conditions, as rural populations remain deprived of adequate amenities in their vicinity. The situation is likely to get further complicated as growing economies also usher in an epidemiological transition to NCDs, driven by affluence accompanied by lifestyle changes. Dealing with screening, preventative care, and long-term management of NCDs while continuing to keep infectious diseases in check will require bold policy initiatives and accelerated execution of infrastructure strengthening.

As the world's second most populous country and its largest lower-middle-income economy, India plays an important role in global progress toward SDG goal 3.8 aspiring to achieve healthcare for all. In addition to its sheer size, the Indian healthcare scenario is complicated by its diverse regional, social, ethnic, cultural, demographic, economic, and political imperatives, all of which must be addressed holistically for UHC programs to be designed and implemented effectively. Over the years, there have been significant efforts invested by the policymakers in strengthening the infrastructure for healthcare delivery, resulting in significant successes and improvements in health outcomes; however, these efforts need to be intensified further to cater to the rapidly increasing population. The increment in SCI at the upper range among all the World Bank income group countries is an impressive achievement, especially in controlling infectious diseases. However, the country still lags on the service capacity front with the sub-index still much below the desired level. There is an unequivocal acknowledgment of the need to rapidly ramp up the infrastructure and skilled workforce as evidenced in recently announced government programs. A strong political will and an integrated approach to overcoming the inherent structural challenges of the system will determine the success of these programs and thus progress toward UHC.
